# Effect of Syzygium cumini and Bauhinia forficata aqueous-leaf extracts on oxidative and mitochondrial parameters in vitro

**DOI:** 10.17179/excli2015-576

**Published:** 2015-11-27

**Authors:** Assis Ecker, Francielli Araujo Vieira, Alessandro de Souza Prestes, Matheus Mulling dos Santos, Angelica Ramos, Rafael Dias Ferreira, Gabriel Teixeira de Macedo, Claudia Vargas Klimaczewski, Rodrigo Lopes Seeger, João Batista Teixeira da Rocha, Nilda B. de Vargas Barbosa

**Affiliations:** 1 Department of Biochemistry and Molecular Biology, Universidade Federal de Santa Maria, 97105-900, Santa Maria, RS, Brazil

**Keywords:** Bauhinia forficate, Syzygium cumini, mitochondria, antioxidant properties

## Abstract

Aqueous-leaf extract of *Syzygium cumini* and *Bauhinia forficata* are traditionally used in the treatment of diabetes and cancer, especially in South America, Africa, and Asia. In this study, we analyzed the effects of these extracts on oxidative and mitochondrial parameters *in vitro*, as well as their protective activities against toxic agents. Phytochemical screenings of the extracts were carried out by HPLC analysis. The *in vitro* antioxidant capacities were compared by DPPH radical scavenging and Fe^2+^ chelating activities. Mitochondrial parameters observed were swelling, lipid peroxidation and dehydrogenase activity. The major chemical constituent of *S. cumini* was rutin. In *B. forficata* were predominant quercetin and gallic acid. *S. cumini* reduced DPPH radical more than *B. forficata*, and showed iron chelating activity at all tested concentrations, while *B. forficata* had not similar property. In mitochondria, high concentrations of *B. forficata* alone induced a decrease in mitochondrial dehydrogenase activity, but low concentrations of this extract prevented the effect induced by Fe^2+^+H_2_O_2_. This was also observed with high concentrations of *S. cumini*. Both extracts partially prevented the lipid peroxidation induced by Fe^2+^/citrate. *S. cumini* was effective against mitochondrial swelling induced by Ca^2+^, while *B. forficata* alone induced swelling more than Ca^2+^. This study suggests that leaf extract of *S. cumini* might represent a useful therapeutic for the treatment of diseases related with mitochondrial dysfunctions. On the other hand, the consumption of *B. forficata* should be avoided because mitochondrial damages were observed, and this possibly may pose risk to human health.

## Introduction

*Syzygium cumini* and *Bauhinia forficata* are tropical plants with worldwide distribution, popularly used in south of Brazil and Asia as antidiabetic agents. These plants have been used for the same medical purpose, despite their properties are still studied separately. *Syzygium cumini* L. belonging to the Myrtaceae family is a tropical evergreen tree (Chanda and Kaneria, 2012[6]). Studies suggest that their leaves extract can reduce radiation-induced DNA damage in cultured cells (Jagetia and Baliga, 2002[13]), is antinociceptive agent to rodents (Quintans et al., 2014[34]) and can prevent the increase of adenosine deaminase activity under hyperglycemic conditions (De Bona et al., 2014[8]). On the other hand, *Bauhinia forficata* (also cited as *Bauhinia forficate* and belonging to the Fabaceae family) is mostly distributed in tropical countries from Africa, Asia and South America. It is traditionally known as “cow's paw”, due to the characteristic bilobed aspect of their leaves (Filho, 2009[10]). Natural constituents from this plant are described as cell-cycle inhibitor as well as inductor of apoptosis in different cell lines, suggesting citotoxicity and possible anticancer properties for this plant (Lim et al., 2006[20]). However, a large number of studies describe especially its hypoglycemic potential (Menezes et al., 2007[22]; Pepato et al., 2002[31]; Silva et al., 2002[37]; Trojan-Rodrigues et at., 2012[40]).

During the treatment of chronic diseases, important cellular functions can be altered, such as metabolism and ATP generation. In this context, mitochondria are potential target to study toxicology and pharmacology of medicinal plants (Zheng et al., 2015[42]). In this context, mitochondrial effects of *S. cumini* and *B. forficata* should be explored. Mitochondria play a pivotal role in cellular functions, including the regulation of apoptosis/necrosis and energy metabolism (Bratic and Trifunovic, 2010[3]). A decrease in mitochondrial viability is a possible indicator of imminent cell death (Kroemer et al., 2007[17]; Orrenius et al., 2015[29]). A range of conditions, including the exposure of mitochondria to exogenous pro-oxidant agents or different diseases, are capable to induce loss in mitochondrial viability and increased reactive oxygen species (ROS) production. These damages are observed in different experimental procedures, including *in vitro* studies (Komulainen et al., 2015[16]; Puntel et al., 2013[33]).

Mitochondria are sensible to little variations in the concentrations of adjacent molecules. As examples, high concentrations of Fe^2+^ and citrate can induce lipid peroxidation (Minotti and Aust, 1987[23]), while elevated concentrations of Ca^2+^ leads to a variety of harmful consequences, such as opening of the permeability transition pores (PTP) and consequent induction of mitochondrial swelling (Andreu et al., 2005[2]). These alterations are related with different pathologies and generally are associated with decreasing in the mitochondrial viability (Orrenius et al., 2015[29]). Lipid peroxidation is related with the impairment between antioxidant defenses and production of ROS, being a common effect in different pathologies involving oxidative stress (Lay et al., 2014[19]; Pillon et al., 2012[32]). At this condition, endogenous antioxidant systems are not able to combat the excessive production of ROS. Consequently, a treatment with exogenous antioxidants and natural products can be a recommendable alternative (Oboh et al., 2007[27]). In this way, it is essential to know the mitochondrial effects of *S. cumini* and *B. forficata* at abnormal oxidative conditions.

Thus, the objective of this study was to compare the general antioxidant capacity of the aqueous leaf extracts of *S. cumini *and *B. forficata*. Furthermore, we aimed to analyze and compare their mitochondrial effects alone as well as their capacity to protect mitochondria against toxic agents. 

## Materials and Methods

### Plant material

#### Extract preparations

*S. cumini* and *B. forficata *were collected from Botanical Garden of Federal University of Santa Maria (UFSM). Voucher specimens of the plants were deposited at the herbarium of UFSM. Thirty grams (30 g) of leaves were maintained in 100 mL H_2_O distilled, at 100 °C, during 30 minutes. After this period, the infusions were filtered and lyophilized. 

#### Quantification of flavonoids and phenolic compounds by HPLC-DAD

Flavonoids and phenolic compounds of extracts were quantified in a high performance liquid chromatography (HPLC) system (Shimadzu, Kyoto, Japan). The HPLC system was equipped with auto sampler, pumps connected to a degasser with integrator, and diode-array UV-VIS detector (DAD). Reverse phase analyses were carried out under gradient conditions using C_18 _column (4.6 mm x 250 mm). The mobile phase consisted in aqueous medium containing 2 % acetic acid (A) and methanol (B). The gradient was composed by 5 % of B until 2 min and posterior changes to obtain 25 %, 40 %, 50 %, 60 %, 70 % and 100 % of B at 10, 20, 30, 40, 50 and 80 min, respectively, following the method described by Laghari et al. (2011[18]) with some modifications. The lyophilized *S. cumini* and *B. forficata* leaves extracts were dissolved in water at 5 mg/mL. The presence of caffeic, chlorogenic and gallic acids (phenolic compounds) and kaempferol, quercetin and rutin (flavonoids) was investigated. The parameters of the HPLC analysis were flow rate of 0.5 ml/min and wavelengths of 325 nm for caffeic and chlorogenic acids, 254 nm for gallic acid and 365 nm for quercetin, rutin and kaempferol. The samples and mobile phase were filtered through 0.45 μm membrane filter and degassed in ultrasonic bath prior to use. Standard solutions of references were prepared in the mobile phase at concentrations of 0.020 - 0.250 mg/ml. The chromatography peaks of the extracts were compared with the commercial standard references in triplicate at room temperature. All the procedures of HPLC-DAD quantification are similar to described by Sousa et al. (2013[38]).

### In vitro experiments

#### DPPH^·^ radical reduction activity

The scavenger capacity or ability of MG to donate hydrogen to 2,2-diphenyl-1-picrilidrazil (DPPH) radical was performed by the method described by Choi et al. (2002[7]). The DPPH reduction was observed spectrophotometrically at 518 nm. In this parameter, aqueous leaf extracts of *B. forficata *were tested at 5, 10, 25, 50, 100, 250, 300, 400 and 500 g/mL and* S. cumini *was tested at concentration of 5, 10, 25, 50, 75, 100; 250 and 500 g/mL. Ascorbic acid, used here as a positive control, was tested at 2, 4, 7 and 10 g/mL. Afterwards, the IC50 values for each extract and for the ascorbic acid were calculated.

#### Fe²^+ ^chelating activity of extracts

The possible iron (Fe²^+^) chelating activity of the extracts was determined by the competitive assay using the indicator 1-10-phenanthroline (*ortho*-phenanthroline) (Klimaczewski et al, 2014[15]). Extracts were tested at concentrations of 5 - 300 g/mL. Solutions of iron sulfate (FeSO_4_) were freshly prepared just before use. FeSO_4_ (at a ﬁnal concentration of 120 µM) was added to tubes containing 0.9 % saline, 0.1 M Tris-HCl, pH 7.5 and the extracts. After incubation (10 minutes at 37 °C), *ortho*-phenanthroline (at a ﬁnal concentration of 0.25 %) was added and the color of the Fe^2+^-phenanthroline complex was determined spectrophotometrically at 510 nm.

### Mitochondrial experiments

#### Animals

Adult male Wistar rats (200 - 250 g) from Central Biotery of Federal University of Santa Maria (UFSM) were maintained in free access to water and food under controlled temperature (22 ± 3 °C) and on a 12 hours light/dark cycle. All the animal manipulation was performed in accordance with the rules of the Brazilian Animal Welfare Committee (Colégio Brasileiro de Experimentação Animal - COBEA), based in principles found in the US guidelines (NIH publication #85-23, revised in 1985).

#### Mitochondrial fraction isolation

Mitochondria were isolated by differential centrifugation, following the methodology described by Brustovetsky and Dubinsky (2000[4]), and Klimaczewski et al. (2014[15]). Protein quantification was performed as described by Lowry et al. (1951[21]).

#### Mitochondrial function/viability

The mitochondrial viability was analyzed by MTT assay, as described by Mosmann (1983[24]) and Franco et al. (2007[11]) with some modifications. Mitochondria (0.3 mg of protein/ml) were pre-incubated with Fe²^+^ (250 µM)/ H_2_O_2_ (1 mM), during 20 minutes at 37 °C. After pre-incubation, mitochondria was incubated 40 minutes at 37 °C in the presence or absence of aqueous leaf extracts of *B. forficata *or* S. cumini, *at concentrations of 5 - 400 µg/mL. Afterwards, 10 μL of MTT solution (5 mg/mL, in ethanol) was added to the medium, and a new incubation was performed for 60 min at 37 °C. The formazan crystals were decanted by centrifugation. Afterwards, the supernatant was discarded and the pellets were dissolved in DMSO for the formazan quantification in a plate reader at 540 nm. Data were expressed as percentage of control. 

#### Mitochondrial swelling

This parameter was measured following the methodology described by Andreu (2005[2]), with some modifications. Mitochondria (0.4 mg of protein/ml) were exposed to CaCl_2_ 50 µM in the presence or absence of *B. forficata *or* S. cumini *extract, at concentrations of 25 - 400 µg/mL. The effects of the extracts alone were also analyzed in this parameter.

#### TBARS production

Thiobarbituric acid reactive species (TBARS) were determined following the method described by Ohkawa et al. (1979[28]) and Klimaczewski et al. (2014[15]) with some modifications. Mitochondria (1.4 mg of protein/mL) were treated with or without Fe^2+ ^(50 µM)/Citrate (2 mM) and with or without aqueous leaf extracts of *B. forficata *or* S. cumini*, by 20 minutes at 37 °C, at concentrations of 5 - 400 µg/mL. After treatments, the TBARS production measurements were performed spectrophotometrically at 532 nm.

#### Statistical analysis

The results were analyzed by one-way ANOVA, following the Tukey post-test. Data were considered statistically different when p < 0.05.

## Results

**Figure 1:**
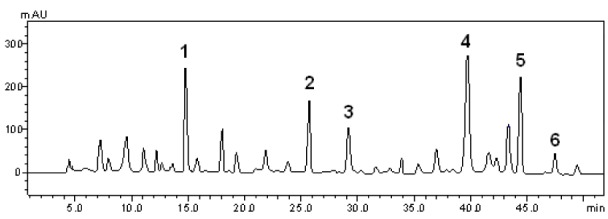
Representative high performance liquid chromatography (HPLC) profile of *Syzygium cumini*, UV detection was at 325 nm. Gallic acid (peak 1), chlorogenic acid (peak 2), caffeic acid (peak 3), rutin (peak 4), quercetin (peak 5) and kaempferol (peak 6) are indicated. Chromatographic conditions are described in the Methods section.

**Figure 2:**
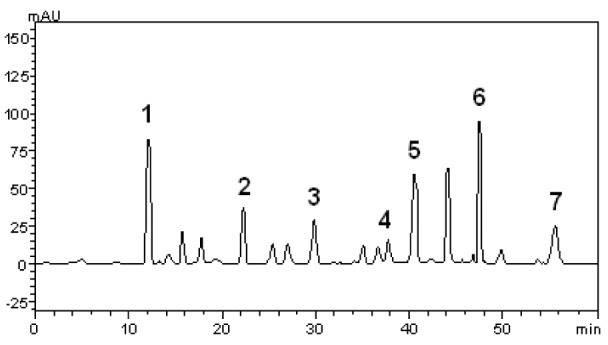
High performance liquid chromatography (HPLC) profile of *B. forficata* extract. Gallic acid (peak 1), chlorogenic acid (peak 2), caffeic acid (peak 3), rutin (peak 4), isoquercitrin (peak 5), quercetin (peak 6) and kaempferol (peak 7) are indicated. Chromatographic conditions are described in the Methods section

The phytochemical constituents of the aqueous leaves extract of the plants were identified by comparisons of the retention times (t_R_) and ultraviolet (UV) spectra of analytical standards analyzed under similar conditions (Figures 1(Fig. 1) and 2(Fig. 2)). The chromatograms of *S. cumini *revealed the presence of gallic acid (3.46 %; t_R_ = 14.72 min; peak 1), chlorogenic acid (2.09 %; t_R_ = 25.81 min; peak 2), caffeic acid (1.57 %; t_R_ = 34.27 min; peak 3), rutin (4.95 %; t_R_ = 39.64 min; peak 4), quercetin (3.37 %; t_R_ = 54.43 min; peak 5) and kaempferol (0.62 %; t_R_ = 47.28 min; peak 6) (Figure 1(Fig. 1) and Table 1(Tab. 1)). In the analysis of the aqueous leaf extract of *B. forficata*, the regions of chromatogram showed typical patterns of UV absorption, supporting the presence of gallic acid (6.53 %, peak 1), chlorogenic acid (2.08 %, peak 2), caffeic acid (1.72 %; peak 3), rutin (0.91 %; peak 4), isoquercitrin (4.45 %; peak 5), quercetin (7.19 %; peak 6) and kaempferol (2.30 %; peak 7), (Figure 2(Fig. 2) and Table 2(Tab. 2)). Therefore, HPLC analysis revealed that hydrolysable tannins, flavonoids and phenolics are the major components of the extract. The calibration curve equations for the constituents were: gallic acid: Y = 15067x + 1020.6 (r = 0.9999); chlorogenic acid: Y = 12569x + 1182.3 (r = 0.9998); caffeic acid: Y = 17483x + 1153.9 (r = 0.9998); rutin: Y = 10361x - 1235.8 (r = 0.9991); quercetin: Y = 15083x - 1341.7 (r =0.9999) and kaempferol: Y = 130745x - 1097.9 (r = 0.9997). 

**Figure 3:**
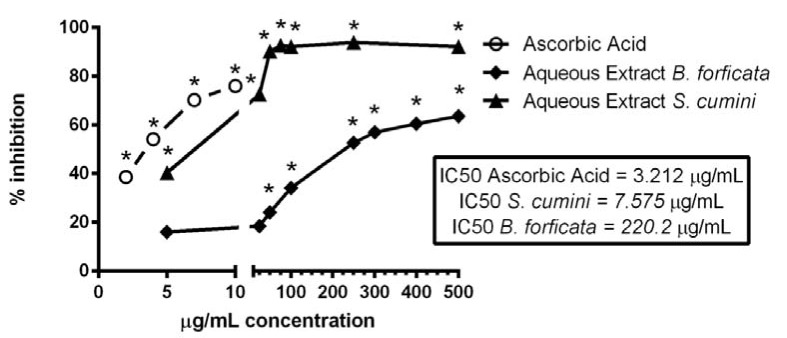
Effect of aqueous leaf extracts of *S. cumini* and *B. forficata* on the DPPH reduction. *B. forficata *was tested at 5, 10, 25, 50, 100, 250, 300, 400 e 500 g/mL. *S. cumini *was tested at 5, 10, 25, 50, 75, 100; 250 and 500 g/mL. Ascorbic acid was used as a positive control, and was tested at 2, 4, 7 and 10 g/mL. * signifies *p <* 0.05 by one-way ANOVA, followed by Bonferroni's post test, when compared with the control inhibition (DPPH without extracts).

The antioxidant capacity of aqueous leaves extract of the plants *S. cumini* and *B. forficata*, at concentrations of 5, 25, 50, 75, 100, 250, 300, 400 and 500 µg/mL, were analyzed by their capacity to reduce the DPPH radical. In this parameter, the results of plants were compared with ascorbic acid, at concentrations of 2, 4, 7 and 10 µg/mL, was used as a positive control (Figure 3(Fig. 3)). All tested concentrations of ascorbic acid and the extracts of *S. cumini* reduced significantly the DPPH radical, indicating high antioxidant capacity. However, aqueous leaf extract of B*. forficata* showed this capacity only at concentrations greater or equal to 50 µg/mL. Therefore, aqueous leaf extract of *S. cumini* showed a better direct antioxidant activity when compared with the same extract of *B. forficata* (the concentrations responsible for the reduction of 50 % of the DPPH radical were 7.575 and 220.2 µg/mL, respectively, while the IC50 value of ascorbic acid was 3.212 µg/mL) (Figure 3(Fig. 3)). Thus, we observed that the *S. cumini* extract was approximately 29 times more effective than *B. forficata* in the reduction of DPPH radical.

**Figure 4:**
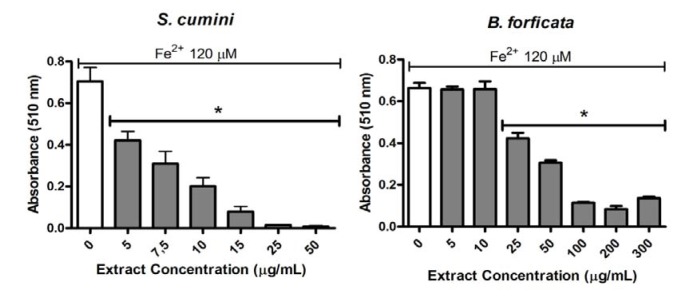
Fe^2+^ chelating activity of *S. cumini *and *B. forficata* aqueous leaves extracts by *ortho*-phenantholine method. Extracts were tested at concentrations of 5 - 300 g/mL together with Fe^2+ ^120 µM. * signify statistically different (*p <* 0.05) from Fe^2+^ without extract (0).

The capacities of the aqueous leaf extract of the *S. cumini* and *B. forficata* to chelate the 120 µM Fe^2+^ were compared by *ortho*-phenantholine method (Figure 4(Fig. 4)). The extracts were analyzed at 5µg/mL or more. However, one more time, *S. cumini* extract was better than *B. forficata* in the Fe^2+^ chelating activity. In this parameter, all tested concentrations of *S. cumini* had a significant effect on this parameter. In relation to *B. forficata* extract, only the largest concentrations showed a significant chelation of 120µM Fe^2+^ (25 µg/mL or more of extract).

**Figure 5:**
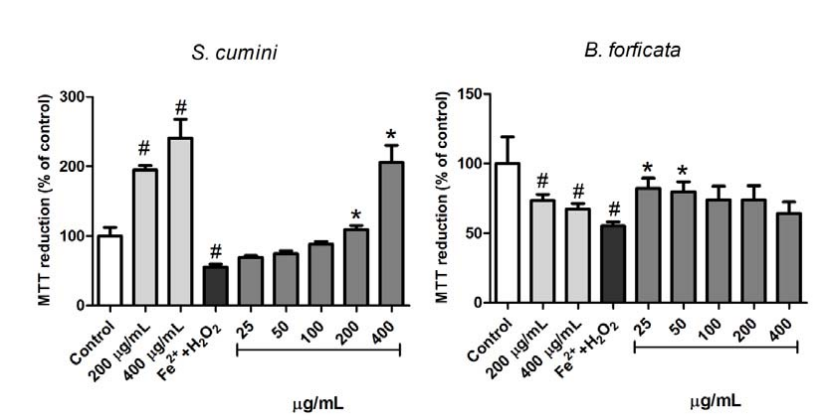
Effects of aqueous leaf extract of *S. cumini* and *B. forficata* on the mitochondrial dehydrogenase activity. Extracts were tested alone or against Fe^2+ ^250µM+H_2_O_2 _1mM (by MTT method). * signifies different from Fe^2+^+H_2_O_2_ (*p* < 0.05) and # is different from Control (*p* < 0.05).

The effects of aqueous leaf extracts of the plants (*S. cumini* and *B. forficata*) against the loss of mitochondrial viability induced by Fe^2+ ^250 µM + H_2_O_2_ 1 mM was analyzed by MTT reduction, as previously described, and the results are exposed in the Figure 5(Fig. 5). In this parameter, an increase in the MTT reduction was observed when the mitochondria were treated with both concentrations of *S. cumini* leaf extract tested alone (200 and 400 µg/mL), indicating a possible increase in the basal metabolism of mitochondria by these concentrations. On the other hand, the same concentrations of aqueous leaf extract of *B. forficata* induced a decrease in the MTT reduction, indicating a damage caused by this extract at high concentrations. The positive control (Fe^2+ ^250 µM + H_2_O_2_ 1 mM) fulfills its role inducing a significant decrease in the MTT viability. However, *S. cumini* and *B. forficata* leaf extracts prevent this effect at higher (200 and 400 µM) and lesser (25 and 50 µM) concentrations, respectively. 

**Figure 6:**
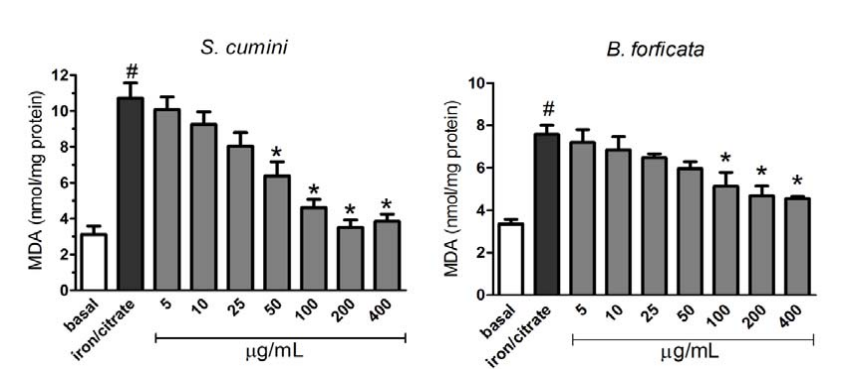
Effect of aqueous leaf extracts of *S. cumini *and *B. forficata *on 50 µM Fe^2+^/citrate-mediated mitochondrial membrane lipid peroxidation in isolated rat liver mitochondria, measured by MDA generation. * signifies statistically different (*p* *<* 0.05) from the Fe^2+^/citrate value, and # is statistically different (*p *< 0.05) from basal value.

The effect of aqueous leaf extracts of the plants (*S. cumini* and *B. forficata*) against mitochondrial lipid peroxidation induced by 50 µM Fe^2+^/citrate are showed in the Figure 6(Fig. 6). Here, 50 µM Fe^2+^/citrate increased significantly the MDA production compared with the basal formation. However, both extracts were able to prevent this effect at higher concentrations. *S. cumini *leaf extract prevented the lipid peroxidation at concentrations greater or equal to 50 µg/mL (first panel of Figure 6(Fig. 6)), while *B. forficata* leaf extract prevented this damage induced by at all concentrations greater or equal to 100 µg/mL (second panel of Figure 6(Fig. 6)). 

**Figure 7:**
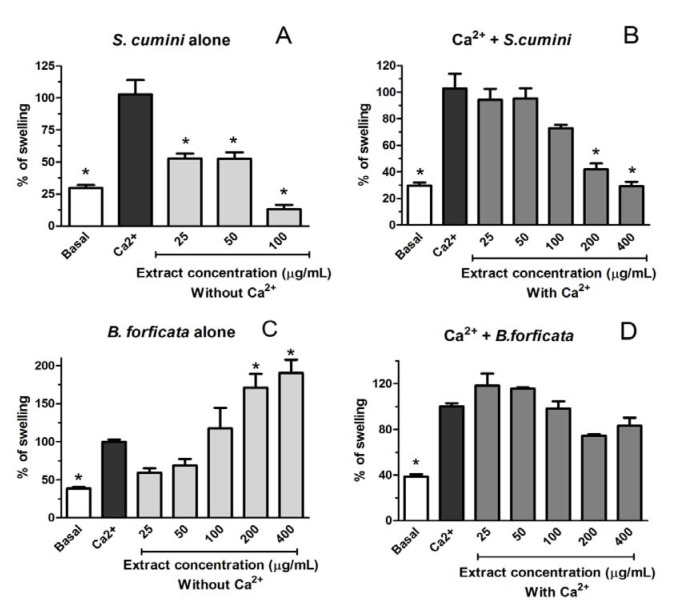
Effects of aqueous extract from leaves of *Syzygium cumini *(A and B) and *Bauhinia forficata *(C and D) on the rat liver mitochondrial swelling with Ca^2+ ^50 µM induction (B and D) or without Ca^2+ ^(50 µM) induction (A and C). Mitochondria were isolated from rat liver. The results are expressed in % of swelling (compared with calcium induction alone). The graph represent the means of 3 independent experiments for each group and * signify statistically different (*p <* 0.05) from calcium alone (Ca^2+^).

For the investigation of the effects of aqueous leaves extract of *S. cumini* and *B. forficata* in different mitochondrial parameters, we analyzed the induction of mitochondrial swelling by both and if they are capable to prevent this damage induced by Ca^2+ ^50 µM (Figure 7(Fig. 7)). Ca^2+^ 50 µM, as expected, induced a significant mitochondrial swelling compared with the control group (Basal). In the same way, aqueous leaf extract of *B. forficata* alone induced a significant mitochondrial swelling in a concentration-dependent manner. The two highest concentrations of this extract alone (200 and 400 µg/mL) caused damage statistically higher than Ca^2+^ 50 µM (Figure 7C(Fig. 7)). Consequently, no tested concentration of this extract was able to prevent the effect induced by Ca^2+^ 50 µM (Figure 7D(Fig. 7)). On the other hand, the tendency to induce mitochondrial swelling was not observed when the mitochondria were treated with aqueous leaf extract of *S. cumini* (Figure 7A(Fig. 7)). Furthermore, this extract prevents the mitochondrial swelling induced by Ca^2+^ 50 µM in a concentration dependent manner (Figure 7B(Fig. 7)), indicating an opposite effect of each plant in this parameter.

## Discussion

The main effort of this work was to perform, for the first time, a comparative study between the effects of aqueous leaf extract from *S. cumini* and *B. forficata* in oxidative and mitochondrial parameters. These plants are largely distributed around the world and are traditionally used in the treatment of pathological conditions, especially *diabetes mellitus* and cancer (Menezes et al., 2007[22]; Pepato, 2002[31]; Lim et al., 2006[20]; Trojan-Rodrigues et al, 2012[40]). However, different properties were observed for each extract among the parameters analyzed. Initially, HPLC analysis showed different profiles between their chemical compositions. Rutin was the major component of *S. cumini*, while quercetin and gallic acid were more abundant in *B. forficata*. The quantitative chemical composition in a significant decreasing order to *S. cumini* was rutin > gallic acid = quercitin > chlorogenic acid > caffeic acid > kaempferol. To *B. forficata*, this sequence was quercitin = gallic acid > isoquercitin > chlorogenic acid = kaempferol > caffeic acid > rutin. Recently, De Bona et al. (2014[8]) suggests that extract of *S. cumini* can be more effective than their individual compounds in the prevention of cellular alterations induced by specific conditions, as hyperglycemia. In this way, the objective of this study was to compare the effects of the extracts in a form that conserve similar characteristics of consumed by general population, and the individual antioxidant or mitochondrial effect of each component identified in HPLC analysis was not considered. 

Flavonoids and phenolic compounds are known to attribute antioxidant characteristic to natural products (Silva et al., 2007[36]). This activity is outstanding in the combat of various complications induced by different agents or pathologies in animal organisms (Aboul-Enein et al.; 2013[1]; Santos et al., 2014[35]). Therefore, extracts with higher antioxidant capacity can be good candidates to assist or even to treat various alterations related with oxidative stress. In this work, a comparison of the *in vitro *antioxidant activities of the extracts was evaluated by their capacities to reduce the DPPH radical and to chelate the Fe^2+^. Here, for the first time, we observed a great difference between the activities of *S. cumini* and *B. forficata*. In relation to reduction of DPPH radical, *S. cumini* extract was approximately 29 times better than *B. forficata*, as indicated by IC50 values. Nevertheless, the standard antioxidant ascorbic acid had an IC50 value still lower than *S. cumini *extract. Thus, our results suggest that *S. cumini* extract can be preferred for the treatment of diseases with oxidative nature. It probably can reduce free radicals and other pro-oxidant agents better than *B. forficata*.

In relation to iron chelating activity, we newly observed differences between the extracts. The most frequent importance to this evaluation is due to the toxic role of iron linked with the catalytic decomposition of hydrogen peroxide via Fenton reaction, leading to ROS formation and causing damage to biomolecules, including lipids, proteins and DNA (Minotti and Aust, 1987[23]). In this way, *S. cumini* aqueous leaf extract decreases the Fe^2+^-phenanthroline complex formation in a concentration-dependent manner. This extract, at concentrations greater or equal to 5 µg/mL, which was the lowest tested concentration for both plants, was capable to chelate significantly 120 µM Fe^2+^. On the other hand, the capacity of *B. forficata* to prevent the formation of Fe^2+^-phenanthroline complex was observed only at concentrations greater or equal to 25 µg/mL, and this was not in a concentration-dependent manner. Consequently, the capacity to prevent oxidative damages via Fenton reaction was observed more effectively in the *S. cumini* extract.

Observing the antioxidant capacity of the extracts we decided to investigate their effects in some mitochondrial parameters. Mitochondria are the most important source of ROS production, especially superoxide (Murphy, 2009[25]). On the other hand, these organelles are responsible to a variety of functions in the most cells (Vliet et al., 2014[41]), including ATP formation through the oxidative phosphorylation. Thus, any mitochondrial damage is related with injuries affected by homeostasis changes in the organisms. To investigate these parameters, the effect of the extracts on the viability, lipid peroxidation and swelling of rat liver mitochondria were analyzed. Decline in the basal activity of their dehydrogenase enzymes are generally related to decreased cell viability, and this effect can be consequence of mitochondrial exposition to metals and ROS (Jomova and Valko, 2011[14]). Nevertheless, increases in the activity of these enzymes are observed at specific conditions, as changing in energy demands or by the presence of specific regulators, such aminoacids or in response to pathologies or senescence (Bratic and Trifunovic, 2010[3]; Passarella et al., 2003[30]). Here, we observed interesting effects of *S. cumini* aqueous leaves extract. In this parameter, *S. cumini* at 200 and 400 µg/mL increased the basal reduction of MTT to formazan by mitochondrial dehydrogenases. As expected, the pro-oxidants agents Fe^2+^ and H_2_O_2_ (Fe^2+^/H_2_O_2_) concomitantly caused a significant decrease in this parameter. However, *S. cumini* extract prevented this damage. On the other hand, similar concentrations of *B. forficata* alone (200 and 400 µg/mL) induced decrease in mitochondrial dehydrogenase activity, in a comparable manner to exposition to oxidative agents. Yet, a concomitant treatment between Fe^2+^/H_2_O_2_ with low concentrations of the *B. forficata* extract (25 and 50 µg/mL) did not differ from the activity of this plant, indicating a possible protective effect of its aqueous leaf extract, at low concentrations, against oxidative damages induced by ROS and metals.

Oxidative damages are generally associated with lipid peroxidation, when hydroxide radical is generated via Fenton's reaction, leading to cellular lipid peroxidation (Minotti and Aust, 1987[23]). Mitochondria are organelles containing two membranes with a well-defined lipid composition, being the most of these lipids synthesized in the endoplasmic reticulum (Tatsuta et al., 2013[39]). However, *de novo* lipid synthesis and remodeling of mitochondrial lipids are important for maintaining the structural integrity and function of mitochondria (Zhong and Yin, 2015[43]). In this work, lipid peroxidation was induced by Fe^2+^ and citrate, concomitantly. As a result, significant increase in MDA levels (a lipid peroxidation metabolite) was observed when mitochondria were exposed only to the agents Fe^2+^ and citrate. However, aquous leaves extract of both plants partially prevented this damage. *S. cumini* extract had protective effect from 50 µg/mL, while *B. forficata* extract was able to prevent the mitochondrial lipid peroxidation only at concentrations greater or equal to 100 µg/mL.

One means by which the mitochondrial-mediated damages occurs in the cells is through the mitochondrial permeability transition (mPT), whereby the inner mitochondrial membrane becomes excessively permeable to ions and other solutes, resulting in a fail of the inner membrane potential, finally leading to energy depletion and cell necrosis (Zoratti and Szabo, 1995[44]; Norenberg and Rao, 2007[26]). The mitochondrial outer membrane is permeable to small solutes and ions, while the inner membrane is virtually impermeable and forms a barrier between the cytosol and mitochondrial matrix. However, Haworth and Hunter (1979[12]) demonstrated that Ca^2+^ can induce mitochondrial swelling, in a phenomenon referred as ''Ca^2+^-induced transition''. This phenomenon is associated with the opening of a proteinaceous permeability transition pore located in the inner mitochondrial membrane. The opening of the pore results in osmotic swelling of the mitochondrial matrix, dissipation of the mitochondrial membrane potential, cessation of the ATP synthesis, and the release of cytochrome c and other factors that leads to apoptosis (Haworth and Hunter, 1979[12]). In this way, mitochondrial swelling is a common parameter of mitochondrial disorder, and can be induced by some exogenous agents, including plants (Cai et al., 2014[5]; Fernandes et al., 2014[9]). In relation to the effects of *S. cumini *and *B. forficata* aqueous leaf extracts, different effects were newly observed. All the effects were compared with the Ca^2+^-induced michondrial swelling. Thus, all the concentrations of *B. forficata* extract alone induced a swelling comparable or higher than Ca^2+^ alone. Furthermore, this extract was not capable to prevent the Ca^2+^-mediated mitochondrial swelling. On the other hand, *S. cumini* extract alone did not induce a mitochondrial swelling compared with Ca^2+^ alone. In addition, the highest tested concentrations of *S. cumini* extract (200 and 400 µg/mL) prevented the swelling induced by Ca^2+^ in a concomitant treatment between mitochondria and extract.

Taken together, the results of this work indicated for the first time that aqueous leaf extract of *S. cumini* and *B. forficata* really can be considered in further studies aiming the treatment of mitochondrial and oxidative damages induced by a variety of pathologies and agents, such diabetes or occupational metal intoxication. However, our results suggested new insights about the differences between the extracts. *S. cumini* was ever more effective than *B. forficata* in the parameters analyzed. Toxicological damage caused by *B. forficata* was observed for a first time (induction of mitochondrial swelling), indicating that further studies are needed to evaluate their systemic effects as well as the limit safe concentration for possible medical use in more complex systems.

## Notes

Assis Ecker and Francielli Araujo Vieira contributed equally to this work.

Araujo Vieira and Rafael Dias Ferreira are deceased.

## Acknowledgements

This work was supported by Brazilian Council of Scientific and Technological Development (CNPq) and Brazilian Coordination for the Improvement of Higher Education Personnel (CAPES). The authors are also thankful to the families of Francielli A. Vieira and Rafael Ferreira for the emotional and intellectual support. This work was idealized by Francielli A. Vieira, deceased in 2013. 

## Conflict of interest

The authors declare that there is no conflict of interest in the conduct and reporting of research.

## Figures and Tables

**Table 1:**
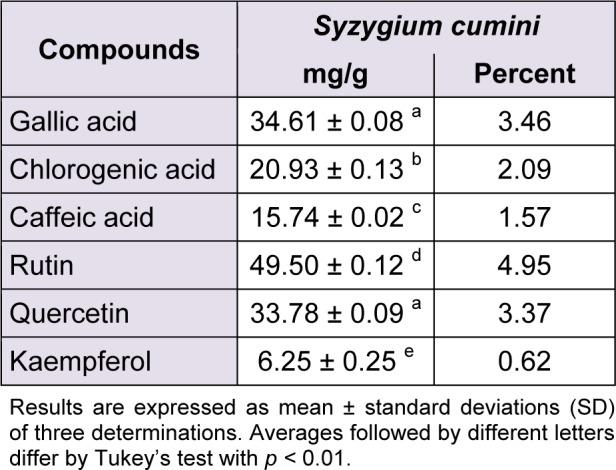
Phenolics and flavonoids composition of *Syzygium cumini*

**Table 2:**
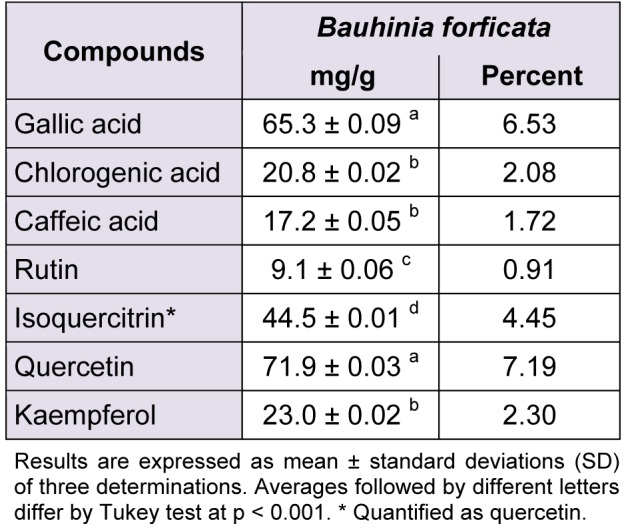
Phenolics and flavonoids composition of *Bauhinia forficata*
